# Fingolimod synergizes and reverses *K. pneumoniae* resistance to colistin

**DOI:** 10.3389/fmicb.2024.1396663

**Published:** 2024-05-30

**Authors:** Xiang Geng, Zhen-Dong Zhang, Yu-Xi Li, Ruo-Chen Hao, Ya-Jun Yang, Xi-Wang Liu, Jian-Yong Li

**Affiliations:** Key Lab of New Animal Drug of Gansu Province, Key Lab of Veterinary Pharmaceutical Development of Ministry of Agriculture and Rural Affairs, Lanzhou Institute of Husbandry and Pharmaceutical Sciences of CAAS, Lanzhou, China

**Keywords:** colistin, fingolimod (FLD), *K. pneumoniae*, antibiotic, synergistic

## Abstract

*Klebsiella pneumoniae* (*K. pneumoniae*) infection and the rapid spread of multi-drug resistant (MDR) bacteria pose a serious threat to global healthcare. Polymyxin E (colistin), a group of cationic antimicrobial polypeptides, is currently one of the last resort treatment options against carbapenem-resistant Gram-negative pathogens. The effectiveness of colistin has been compromised due to its intensive use. This study found that fingolimod (FLD), a natural product derivative, exhibited a significant synergistic bactericidal effect on *K. pneumoniae* when combined with colistin, both *in vitro* and *in vivo*. The checkerboard method was employed to assess the *in vitro* synergistic effect of FLD with colistin. FLD enhanced the susceptibility of bacteria to colistin and lowered effectively minimum inhibitory concentrations (MIC) when compared to colistin MIC, and the fractional inhibitory concentrations (FIC) value was less than 0.3. The time-kill curve demonstrated that the combination treatment of FLD and colistin had significant bactericidal efficacy. The *in vitro* concurrent administration of colistin and FLD resulted in heightening membrane permeability, compromising cell integrity, diminishing membrane fluidity, and perturbing membrane homeostasis. They also induced alterations in membrane potential, levels of reactive oxygen species, and adenosine triphosphate synthesis, ultimately culminating in bacterial death. Moreover, the combination of FLD with colistin significantly influenced fatty acid metabolism. In the mouse infection model, the survival rate of mice injected with *K. pneumoniae* was significantly improved to 67% and pathological damage was significantly relieved with combination treatment of FLD and colistin when compared with colistin treatment. This study highlights the potential of FLD in combining with colistin for treating infections caused by MDR isolates of *K. pneumoniae*.

## Introduction

The persistent utilization of antibiotics resulted in a rise in antibiotics resistance among bacterial pathogens, posing a significant global health challenge (Parmar et al., 2018). An expending array of infections, including pneumoniae, tuberculosis, and salmonellosis, are becoming increasingly difficult to treat due to reduced efficacy of standard antibiotics therapies (Shaaban et al., 2020). Although multidrug resistant (MDR) in both Gram-positive and Gram-negative bacteria was prevalent, the pace of novel drug development has been slower than anticipated (Livermore, 2012; Woolhouse and Farrar, 2014). Considering the challenge posed by the resistance of bacterial pathogens to conventional antibiotics and the limited development of new antibiotics, it is imperative to explore alternative strategies to combat the crisis (Giannella et al., 2020). One of the best strategies is for the development of innovative drug combinations to increase eradication rates and reduce antibiotic resistance, particularly against Gram-negative bacteria. The potential efficacy of combining existing drugs shows promise in mitigating the global crisis of antibiotic resistance and prolong the effectiveness of the existing antibiotics (Zheng et al., 2018; Janardhanan et al., 2019).

**Figure 1:**
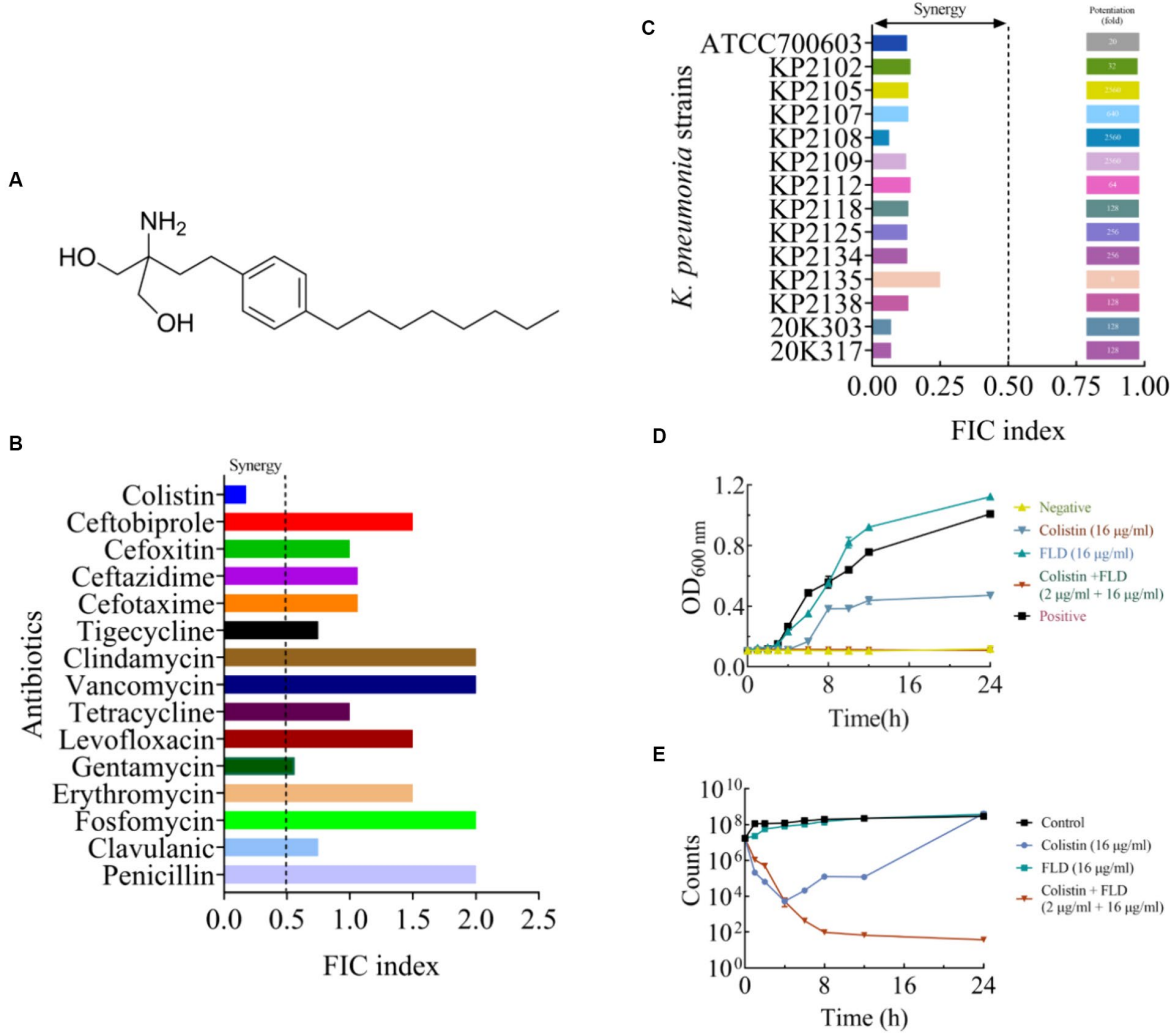
The synergistic effect of FLD in combination with colistin against *K. pneumoniae.* **(A)** The structure of FLD. **(B)** The effect in combination of FLD and various antibiotics against *K. pneumoniae* ATCC 700603. **(C)** FIC indices of the combination between colistin and FLD against standard and clinical *K. pneumoniae* were tested using chequerboard microdilution assays. Synergy is defined as an FIC index ≤0.5. **(D)** Growth curve of KP2108 in the presence of colistin (16 μg/mL), FLD (16 μg/mL), and colistin (2 μg/mL) combination with FLD (16 μg/mL). **(E)** Time-kill curve KP2108 in the presence of colistin (16 μg/mL), FLD (16 μg/mL), and colistin (2 μg/mL) combination with FLD (16 μg/mL).

Colistin is considered a ‘last-resort’ antibiotic for treating infections caused by MDR Gram-negative bacteria, and resistance to colistin can cause difficult or impossible-to-treat infection (Hussein et al., 2022). After binding to lipopolysaccharide (LPS) of Gram-negative bacterial outer membrane, colistin formed a complex with LPS. This complex disrupted the stability of the outer membrane, and followed by disruption of the membrane via replacement of divalent cations such as Ca^2+^ or Mg^2+^ from the membrane lipids, which lead to the leakage of cellular contents and ultimately to death of the bacteria (Saikia et al., 2022). Regrettably, colistin was being increasingly used in clinical practice and uncontrollably in agriculture alone, contributing to the rapid dissemination of resistance (Macnair et al., 2018). Therefore, antibiotics combined with a synergist is feasible and cost-effective solution to the access problem (Xu et al., 2022). Synergists act as adjuvants, enabling antibiotics to function in synergy through various mechanisms including efflux pump inhibition, inhibition of enzymes, and changes in membrane permeability, all of which may contribute to increasing the efficacy of a specific antibiotic (Douafer et al., 2019). At present, antibiotics or antibiotics adjuvants have been extensively explored in various areas, including natural plant (Douafer et al., 2019), chemical synthesis (Song et al., 2020), marine (Zhang et al., 2021), and actinomyces (Genilloud, 2017). Fingolimod (FLD) is a sphingosine analog, which was originally discovered by chemical modification of myriocin (Fujita et al., 1994). FLD (Figure 1A) is approved by the Food and Drug Administration (FDA) for the treatment of multiple sclerosis (Pino et al., 2019), which elicits lymphopenia resulting from a reversible redistribution of lymphocytes from circulation to second lymphoid tissues (Liu et al., 2008). The antibacterial activity of FLD on Gram-positive bacteria, such as *Staphylococcus aureus* (*S. aureus*) and *Staphylococcus epidermidis* (*S. epidermidis*) was confirmed. However, the antibacterial activity of FLD on Gram-negative bacteria was not yet found (Gilbert-Girard et al., 2020). *K. pneumoniae* is a ubiquitous Gram-negative environment bacterium and poses a serious and growing public health threat globally (Horesh et al., 2020). This study aimed to investigate the potential of FLD as an adjuvant drug for colistin to treat infections caused by *K. pneumoniae*.

In this study, FLD significantly improved *K. pneumoniae’*s sensitivity to colistin (FIC<0.5) (Figure 1B), and two combinations produced excellent bacteriostatic and bactericidal effects, include sensitive strain, MDR strain, and hypervirulent strain. Further studies revealed that colistin combined with FLD had strong effects on cell membrane, oxidative stress, and the fatty acid metabolism. It was important to note that FLD effectively enhanced the antibacterial activity of colistin in mouse models of *K. pneumoniae* infection. These results showed that FLD is a potential candidate adjuvant for colistin.

## Materials and methods

### Strains and materials

The FLD was purchased from YuanYe Bio-Technology Co., Ltd. (Shanghai, China) and the purities were above 98%. Colistin, tetracycline, tigecycline, cefoxitin, vancomycin, cephalothin, ceftazidime, cefotaxime, clarithromycin, levofloxacin, gentamicin, erythromycin, ampicillin, fosfomycin, clavulanic acid, and penicillin were obtained from Sigma-Aldrich. The strain of *K. pneumoniae* ATCC 700603 was obtained from American Type Culture Collection (ATCC, Manassas, VA, United States). Clinic strains of *K. pneumoniae* were given from Researcher Xu Chunyan (Henan Agriculture University, CHN), and all strains were cultured in Mueller Hinton (MH) broth medium. MH agar and broth was supplied from Beijing Solarbio Science & Technology Co., Ltd. Unless stated, all *K. pneumoniae* strains were routinely grown in MH broth at 37°C. Stock solutions of all compounds were prepared in DMSO and stored at −20°C.

### Determination of minimum inhibition concentrations (MICs)

The MICs of FLD and antibiotics were determined using the standard broth microdilution method with Mueller Hinton (MH) broth (Clinical and Laboratory Standards Institute, 2021). A single colony was picked and incubated in MH broth at 37°C with 200 rpm overnight, then the bacterial solution was diluted to 10^6^ CFU/mL with a fresh medium. 0.1 mL diluted bacterial solution (10^5^ CFU) and 0.1 mL FLD or antibiotics solution (0.0125 to 6.4 μg) were mixed in U-bottomed 96-well plates, and MICs were recorded after co-culturing for 18 ± 2 h. The final DMSO concentration in MH broth was no more than 1%.

### Drug combination assay

The checkerboard assay was used to determine the synergistic effect of FLD and antibiotics on *K. pneumoniae* as reported (Li et al., 2023). Two-fold series dilutions of antibiotics were mixed with FLD in 96-well plates. 0.1 mL of *K. pneumoniae* solution (10^5^ CFU) were added, and incubated at 37°C for 18 ± 2 h. The fractional inhibitory concentrations (FICs) index between FLD and antibiotics were calculated according to the Formula (1):


(1) $$\mathrm{FICindex}={\mathrm{MIC}}_{\mathrm{AB}}/{\mathrm{MIC}}_{A}+{\mathrm{MIC}}_{\mathrm{BA}}/{\mathrm{MIC}}_{B}={\mathrm{FIC}}_{A}+{\mathrm{FIC}}_{B}.$$

MIC_A_ is the MIC of compound A alone; MIC_AB_ is the MIC of compound A in combination with compound B; MIC_B_ is the MIC of compound B alone; MIC_BA_ is the MIC of compound B in combination with compound A; FIC_A_ is the FIC of compound A; FIC_B_ is the FIC of compound B. The synergy or additive was defined according to standard criteria (FICI ≤0.5 was defined as synergistic; 0.5 < FICI ≤1 was defined as additive; 1 < FICI ≤4 was defined as indifference; FICI >4 was defined as antagonism).

### Growth curve

KP2108 was cultured to an OD_600_ ≈ 0.5 and diluted to 10^6^ CFU/mL. Different concentrations of colistin, FLD, and colistin combined with FLD were added to 5 mL diluted bacterial broth. The bacterial growth was conducted at different time points by recording absorbance with Multiskan™ GO Microplate Reader (Thermo Fisher Scientific).

### Time-kill assay

KP2108 was cultured overnight and diluted to 10^7^ CFU/mL (4 mL). Then strain was incubated with colistin, FLD, combination of colistin and FLD, at 37°C with shaking at 200 rpm. At different time points, 100 μL bacterial solution of each group were collected, diluted in tenfold, and plated onto MH agar to count bacterial numbers after incubation at 37°C for 24 h.

### Bacterial viability assay

To assess cell viability, the live and dead bacteria were visualized by using BacLight bacterial viability assay kit (Invitrogen™) after colistin and/or FLD treatment. KP2108 was cultured overnight, washed, and resuspended in sterile PBS. Bacteria cells with approximate 10^9^ CFU in 0.5 mL solution were incubated for 1 h at 37°C with shaking at 200 rpm, with colistin, FLD, or combination of colistin and FLD. The cells were then washed by centrifugation and resuspended again for staining. After incubation at room temperature for 15 min, photographs were taken using confocal laser scanning microscope (CLSM, LSM800, Zeiss, Jena, Germany).

The flow cytometry samples preparation processes were performed in accordance with the above preparation method of CLSM samples. The samples were subjected to the flow cytometry (Beckman Coulter CytoFLEX-LX). FlowJo v10.8.1 software was used for data analysis and presentation.

### SEM observation

Morphology of bacterial cells was observed by scanning electron microscope (SEM). The cells were harvested after treatment with colistin, FLD, or combination of colistin and FLD for 1 h, and performed by incubation in glutaraldehyde solution (2.5%) at room temperature for 2 h. Then, the cells were exposed to an ethanol series (30, 50, 70, 80, 90, 95, and 100%) for dehydration followed by drying with critical point dryer. Subsequently, samples were sputter-coated with gold, and imaged at different magnifications (HITACHI, SU8100).

### Outer membrane (OM) permeability assay

The outer membrane permeability after treatment with colistin and FLD was assessed by using fluorescent probe 1-*N*-phenylnaphthylamine (NPN). Briefly, KP2108 was cultured overnight, washed, and resuspended in PBS at OD_600_ = 0.5. Then, the probe NPN was added to a final concentration of 1 μM and incubated at 37°C. After incubation for 30 min, 190 μL of NPN-label bacterial liquid and total 10 μL colistin combined with FLD were added per well in a 96-well plate. Immediately, the fluorescence intensity was measured with a multifunctional microplate reader.

### Inner membrane integrity assay

KP2108 single colony was inoculated into MH broth and cultured overnight at 37°C and 200 rpm. The bacterial suspension was then washed twice and resuspended in PBS, and 5 mL bacterial liquid was adjusted to OD_600_ = 0.5. This was followed by the subsequent addition of colistin, FLD, and the combination of both. Culturing was continued at 37°C and 200 rpm and sampled at 0, 1, 2, and 3 h. Next, samples were incubated with propidium iodide (PI, 5 μM) for 20 min at 37°C. After incubation, unbound PI was removed by washing the samples with PBS and the multifunctional microplate reader was used to detect the fluorescence intensity.

### PMBN enhanced FLD antibacterial effect

The membrane-permeabilizing agent polymyxin B nonapeptide (PMBN) was used to investigate whether the lack in bactericidal effect of FLD is attributed to its inability to penetrate the outer membrane. Briefly, 180 μL of MH broth was added to the first row, and 100 μL of MH broth was added to the remaining rows in a 96-well plate. Twenty microliter of FLD stock solution was added to the first row and diluted using twofold dilution to obtain concentration of 0.125 to 64 μg/mL. Next, 5 μL PMBN solution was added to different lines to obtain concentration of 10 μg/mL. Finally, 95 μL KP2108 solution (10^6^ CFU/mL) was added and incubated at 37°C for 18 ± 2 h.

### Membrane fluidity assay

KP2108 was grown overnight from a single colony in MH broth at 37°C and 200 rpm. Bacterium solution was then washed twice and resuspended in PBS, and the bacterial liquid was adjusted to OD_600_ = 0.5. Next, the Laurdan probe was added to the solution at 10 nM and incubated for 30 min at 37°C. This was followed by the subsequent addition of colistin, FLD, and the combination of both, and benzyl alcohol (BZ) was used as a positive control. The excitation wavelength of 350 nm and emission wavelength of 435 nm and 510 nm were used for detection when the samples were protected from light and incubated for 30 min at 37°C. The Laurdan generalized polarization (Laurdan_GP_) was calculated using the following Formula (2):


(2) $${\mathrm{Laurdan}}_{\mathrm{GP}}=\left(I_{435}–I_{510}\right)/\left(I_{435}+I_{510}\right).$$

### Cytoplasmic membrane depolarization assay

The cytoplasmic membrane depolarization assay was conducted according to the previously reported method with slight modification (Cheng et al., 2014). A membrane potential-sensitive fluorescent dye as 3,3’-Dipropylthiadicarbocyanine iodide [DiSC_3_(5)] was used to evaluate the cytoplasmic membrane depolarization activity of colistin combined with FLD. Briefly, the MDR bacteria, KP2108, was incubated at 37°C to an OD_600_ of 0.5, the cells were harvested, washed, and resuspended in HEPES buffer (2 mM HEPES, 5 mM glucose). Subsequently, DiSC_3_(5) was added to the bacterial suspension to 1 μM, which was incubated at 37°C for 20 min. A 190 μL aliquot of cell suspension was transferred into a black 96-well plate and colistin and/or FLD were added to a total volume of 10 μL. A blank with only cell suspension and dye was used for background subtraction. The plate was incubated for 60 min at 37°C and was examined after incubation with compounds. The fluorescence intensity was monitored at an excitation wavelength of 622 nm and an emission wavelength of 670 nm. The fluorescence leakage (*F*_L_) was defined by the following Formula (3):


(3) $$F_{L}=\left(F_{F}–F_{B}\right)–\left(F_{I}–F_{B}\right).$$

*F*_F_: the final fluorescence intensity in assay medium after treatment with antibiotic for 30 min;

*F*_I_: the initial fluorescence intensity of the cell suspension;

*F*_B_: the fluorescence intensity of the blank.

### ΔpH assay

The ΔpH was determined by the pH-sensitive fluorescence probe 2′,7′-bis(2-carboxyethyl)-5(6)-carboxyfluorescein acetoxymethyl ester (BCECF-AM). KP2108 was cultured overnight, then washed, and resuspended in PBS. The fluorescent probe, BCECF-AM, was added into a final concentration of 0.1 μM after the bacterial suspension concentration was adjusted to OD_600_ = 0.5. Then, incubation was implemented for 30 min at 37°C. 10 μL solution of colistin, FLD and combination of both were mixed with 190 μL of bacterial suspension, and the fluorescence value was detected immediately by a multi-purpose microplate reader, with the excitation wavelength at 488 nm and the emission wavelength at 535 nm.

### Reactive oxygen species (ROS) measurement

The overnight cultured bacterial strain KP2108 was washed with PBS and adjusted to concentration at OD_600_ = 0.5. The probe, 2′,7′-dichlorofluorescein diacetate (DCFH-DA), was added to a final concentration of 1 μM, and incubated at 37°C for 20 min. After staining, cells were washed and resuspended, and 190 μL bacterial solution was taken and mixed with 10 μL solution of colistin, FLD, and combination of both. After incubating for 10 min, detection of fluorescence intensity was performed using a multi-purpose microplate reader.

### ATP assay

The Enhanced ATP Assay Kit (Beyotime) was used to determine the extracellular and intracellular ATP levels of KP2108 after being treated with colistin, FLD, and combination of both. Overnight culture of KP2108 was centrifuged and the pellet was resuspended in PBS to an OD_600_ of 0.5. Then, different treatments were mixed with bacterial suspension into 1 mL and the mixed solution was incubated for 20 min. After centrifuging at 4°C with 10,000 × *g*, the supernatants were harvested and used to test extracellular ATP levels. Next, the supernatant was collected after precipitation of the cell lysate with lysis buffer, and the intracellular ATP levels of bacteria were measured with a multi-purpose microplate reader in the model of luminescence.

### Metabolomics analysis

#### Sample preparation

KP2108 metabolite extraction was conducted as previously described with slight modifications (Ye et al., 2021). A single colony was picked and incubated at 37°C overnight. Aliquots were diluted 1:25 in 100 mL fresh MH broth and growth at 37°C on a rotary shaker at 200 rpm until the OD_600_ indicated that the culture reached the mid-exponential phase (approximately 0.35). Colistin combined with FLD were added to the bacterial solutions and an equal concentration of colistin was added to the control group. After 1 h incubation, the bacterial concentration was adjusted to the same concentration with MH broth. Ten microliter aliquots of bacterial cultures were transferred to centrifuge tube containing 40 mL of methanol/ethylene glycol (45:55, *v/v*) precooled to −20°C, respectively. The solution was mixed and centrifuged for 50 min at 4,500 × *g*, −4°C, the supernatant was discarded, and 1 mL sodium chloride solution (85%) was added. Subsequently, the precipitate was resuspended and cleared by centrifugation for 10 min at 20,000 × *g* and 4°C. The supernatant was discarded again, and the tube was tightly capped after 500 μL 75% ethanol was added. The samples were heated at 95°C for 5 min in a metal bath and then extraction/centrifugation was repeated three times. The filtrate was evaporated to dryness at 40°C in a rotary evaporator and redissolved in 500 μL acetonitrile/water (50:50, *v/v*). After centrifugation for 10 min at 20,000 × *g*, 4°C, the supernatant was filtered with a microporous membrane (0.22 μm) before being analyzed by UHPLC-TOF/MS.

### UHPLC-TOF/MS conditions

Intracellular metabolites were analyzed using Agilent 1,290 ultra-performance liquid chromatography equipped with Q-Exactive Orbitrap Plus. Chromatographic separation of metabolites was performed on a Wates T3 (21 mm × 50 mm, 18 μm), the column temperature was set at 50°C, and analysis was performed in both negative and positive ion modes. The mobile phase consists of A (0.1% formic acid aqueous) and B (acetonitrile) by gradient elution. Samples injection volume was 5 μL. The MS conditions were as followed: the electrospray ionization source (ESI) operating in positive and negative ion modes was 3.0 kV, MS/MS resolution as 17,500, the normalized collision energy (NCE) was 25 eV, and the ionic signal was collected in the 100–1,000 m/z range.

### Metabolomics data analysis

The original data were processed using MS-DIAL (version 4.00, http://prime.psc.riken.jp/Metabolomics_Software/MS-DIAL/index2.html) to filter noise, baseline comparison, peak identification, data reduction, and normalization. The two-dimensional data matrix including mass-to-charge ratio, peak area and retention time were obtained. The obtained data were subjected to multivariate statistical analysis using SIMCA-P software (version 14.1), and the results were presented as principal component analysis (PCA) score plots and partial least squares discriminant analysis (OPLS-DA) score plots, respectively. The OPLS-DA model was also evaluated using the *R*^2^*X*, *R*^2^*Y*, and *Q*^2^ parameters, and the permutation test was used to prevent overfitting of the model. The metabolites with VIP > 1 and *p* < 0.05 were screened for metabolite variability analysis in different groups. According to the information on the differential metabolites, MetaboAnalyst 4.0 and KEGG database were used for the analysis of relevant metabolic pathways.

### Molecular docking and molecular dynamics simulation

The LpxC protein crystal structure of *E. coli* (PDB: 4MDT) was downloaded from Protein Data Bank.^1^ Since no crystal structure of LpxC for *K. pneumoniae* (KPLpxC) has been deciphered yet, the SWISS-MODEL^2^ was used to predict the three-dimensional structure of this protein (Waterhouse et al., 2018).

Molecular docking of FLD into the KPLpxC was carried out using the Discovery Studio (version 4.0) as implemented through the graphical user interface DS-CDOCKER protocol. Three-dimensional (3D) structure of FLD was constructed using Chem. 3D ultra 19.0 software (Chemical Structure Drawing Standard; Cambridge Soft Corporation, United States). For protein, the water and impurities were removed and the hydrogen atoms were added. The 3D structure of KPLpxC was placed during the molecular docking procedure. Types of interactions of the docked protein with KPLpxC were analyzed after molecular docking. Compound would retain 10 poses, and were ranked and selected by -CDOCKER_ENERGY.

In order to visualize the binding mode of FLD and KPLpxC, the structural of complex KPLpxC-FLD was further subjected to Molecular dynamics (MD) simulation in a solution system for 100 ns, using the Desmond/Maestro non-commercial (version 2023.21) (Bowers et al., 2006; Shaw, 2022). TIP3P water molecules were added to the systems, which were then neutralized by 0.15 M NaCl solution. After minimization and relaxation of the system, the production simulation was performed for 100 ns in an isothermal-isobaric ensemble at 300 K and 1 bar. Trajectory coordinates were recorded every 100 ps. The molecular dynamics analysis was performed using Simulation Interaction Diagram from Desmond.

### Mouse infection models

A mouse model of endonasal pulmonary infection was used to determine the synergistic effect of FLD in combination with colistin *in vivo*. Animal experiments were approved and conducted in accordance with the guidelines of the Animal Experimental Ethical Committee of Lanzhou institute of husbandry and pharmaceutical science of CAAS (2023–017). All mice were maintained with a maximum of five mice per cage and housed adaptively for 5 days prior to the beginning of the experiment. 20 K317 was cultured to an OD_600_ value at 0.4 in MH broth at 37°C. Eight-week-old female BALB/c mice were randomly divided into 5 groups (blank control, solvent control, colistin, FLD, and colistin combination with FLD) with six mice per group. The mice were lightly anesthetized with an intraperitoneal injection of sodium Ulatan (TIC) as 750 mg/kg b.w. In the experimental groups, the mice were in the position of head up and upright, and 20 K317 was dropped into the nasal cavity for 50 μL (approximate 10^7^ CFU). The same amount of saline was dropped into the nasal cavities in the blank control group. After 2 h, the infected mice were intraperitoneal injection with solvent, colistin (0.5 mg/kg), FLD (30 mg/kg), a combination of colistin (0.5 mg/kg) and FLD (8 mg/kg), and continuously once each day for 5 days. Mice were monitored until day 5 post-infection. Once the infection mice died, different organs, including the heart, liver, spleen, lung, and kidney, were removed for loading bacterial and histological analysis.

### Statistical analysis

The results of all experiments were presented as the mean ± SD for three replicates. Statistical significance was analyzed by GraphPad Prism 8.0 software (GraphPad Software, San Diego, CA, United States) with unpaired Student’s *t*-test or non-parametric one-way ANOVA. Significant values are represented by an asterisk: ^*^p < 0.05, ^**^p < 0.01, ^***^p < 0.001, ^****^p < 0.0001.

## Results

### FLD showed a synergistic effect in combination with colistin against *K. pneumoniae*

The MIC and potential adjuvant effect of FLD and 16 antibiotics against *K. pneumoniae* ATCC700603 was evaluated. The antibiotic tested in this study represented a wide range of antimicrobial classes. FLD showed no activity against *K. pneumoniae* ATCC700603 (MIC >64 μg/mL). However, the synergy between FLD and colistin was observed with a synergistic index of 0.175 (Table 1). Colistin is considered a last resort drug in the treatment of multidrug-resistant (MDR) and extensively drug resistant (XDR) Gram-negative bacterial infections. The MIC of colistin or FLD against KP2108 was greater than 64 μg/mL. Therefore, the combination of colistin with FLD against *K. pneumoniae* is warrants for further investigation. Subsequently, their synergism was evaluated on clinical *K. pneumoniae* strains. The results demonstrated that FLD uniformly increased the sensitivity of all *K. pneumoniae* strains to colistin, with FIC values consistently below 0.5 (Figure 1C). Among the test strains, KP2108 exhibited the most pronounced synergistic effects with the two-drug combination and was therefore selected for subsequent experiment. Based on the checkerboard assay results and sensitivity enhancement of FLD, the ratio of drugs in the combination was selected as colistin (2 μg/mL) and FLD (16 μg/mL).

**Table 1:** Scan for synergistic combination of FLD and various antibiotics against *K. pneumoniae* ATCC 700603.

Compound	MIC (μg/ml)	MIC_combination_ (μg/ml)		FIC index (FLD and antibiotics)
		Antibiotics	Fingolimod	
Colistin	0.5	0.025	8	0.175
Ceftobiprole	>64	32	64	1.5
Cefoxitin	32	16	32	1
Ceftazidime	64	32	4	1.0625
Cefotaxime	16	16	4	1.0625
Tigecycline	2	1	16	0.75
Clindamycin	>64	>64	>64	–
Vancomycin	>64	>64	>64	–
Tetracycline	8	4	32	1
Levofloxacin	0.25	0.125	64	1.5
Gentamycin	8	4	4	0.5625
Erythromycin	64	32	64	1.5
Fosfomycin	>64	>64	>64	–
Clavulanic	32	16	16	0.75
Penicillin	>64	>64	>64	–
Fingolimod	>64			

–, not calculated.

The growth curve result for KP2108 demonstrated that FLD (16 μg/mL) was unable to inhibit bacterial growth, and colistin (16 μg/mL) could partially inhibit bacterial growth (Figure 1D). However, when colistin (2 μg/mL) was combined with FLD (16 μg/mL), a significant suppression of cell growth was observed and the sensitization of FLD was highlighted (Figure 1D). Time-killing assays were conducted to demonstrate the bactericidal ability of the colistin (2 μg/mL) and FLD (16 μg/mL) combination over a 24-h period (Figure 1E).

### FLD combined with colistin disrupts cell viability and morphology

**Figure 2:**
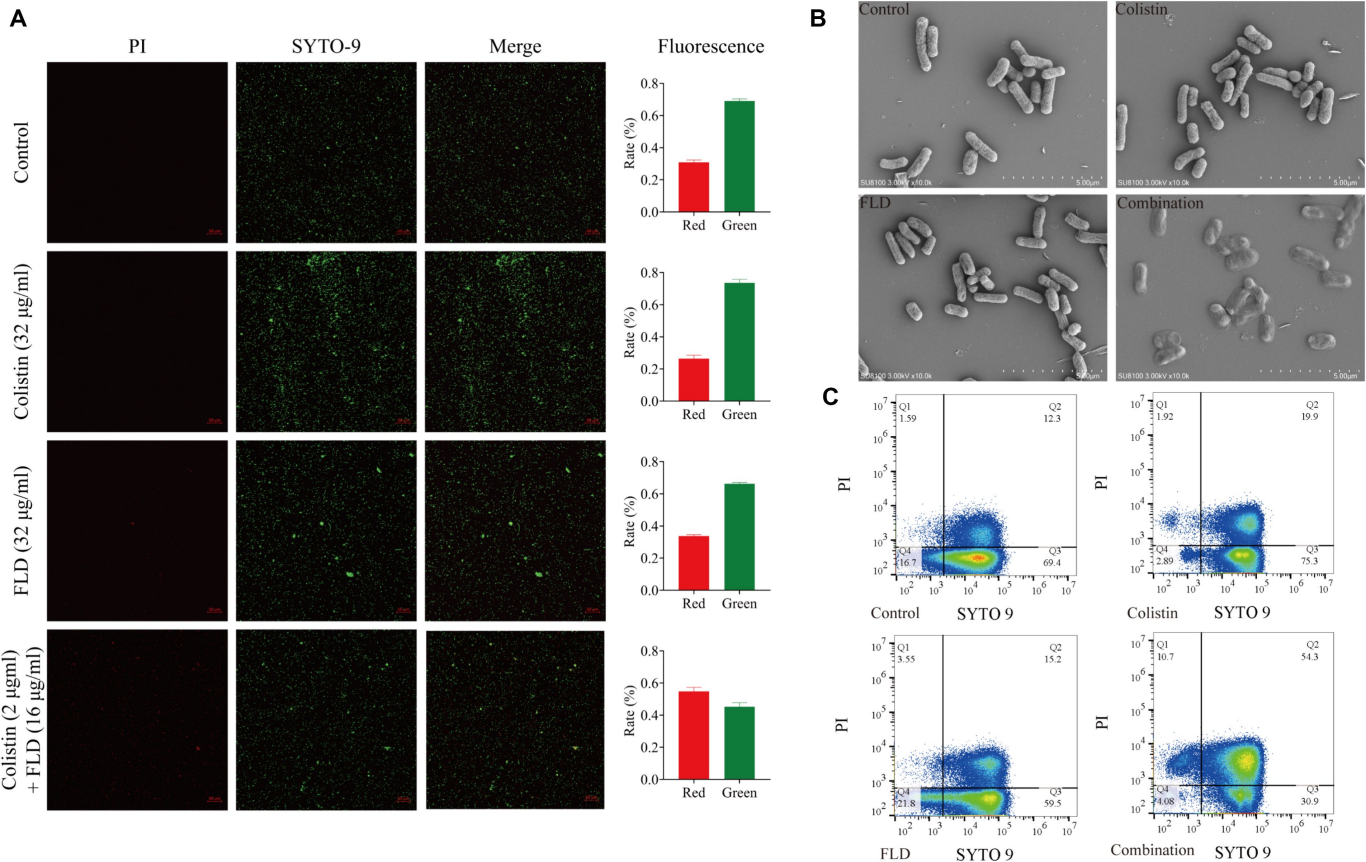
Fingolimod combined with colistin disrupted cell viability and morphology. **(A)** CLSM images of KP2108 under the treatment of Colistin (32 μg/mL), FLD (32 μg/mL), and both combination (2 μg/mL + 16 μg/mL) for 1 h. Viable bacterial cells were stained green by SYTO 9, whereas dead cells were stained red by PI. **(B)** SEM images of KP2108 under the treatment of Colistin (32 μg/mL), FLD (32 μg/mL), and both combination (2 μg/mL + 32 μg/mL) for 1 h. **(C)** Quantified results of the flow cytometric analysis after treated with Colistin (32 μg/mL), FLD (32 μg/mL), and both combination (2 μg/mL + 16 μg/mL) for 1 h.

Following treatment with colistin (32 μg/mL), FLD (32 μg/mL), and the combination of both (2 μg/mL + 16 μg/mL), cells were subjected to staining and visualization using CLSM, SEM, and flow cytometry. The CLSM results demonstrated that the viability of cells treated with colistin or FLD had similar ratio of green-stained cells when compared with the control group. However, the combination of colistin and FLD led to cell death with increase in the ratio of red fluorescent and a decrease in green fluorescent (Figure 2A). SEM analysis revealed a completely collapse of the bacteria structure, with the absence of detectable rod-shaped bacteria, indicating complete disintegration and loss of tridimensional structure following treatment with the colistin and FLD combination (Figure 2B). Similarly, flow cytometric analysis results aligned with the CLSM findings, indicating outer membrane permeability increase and inner membrane disruption, as evidenced by elevated uptake of PI following treatment with both combinations (Figure 2C).

### FLD targets bacterial cytoplasmic membrane against MDR *K. pneumoniae*

**Figure 3:**
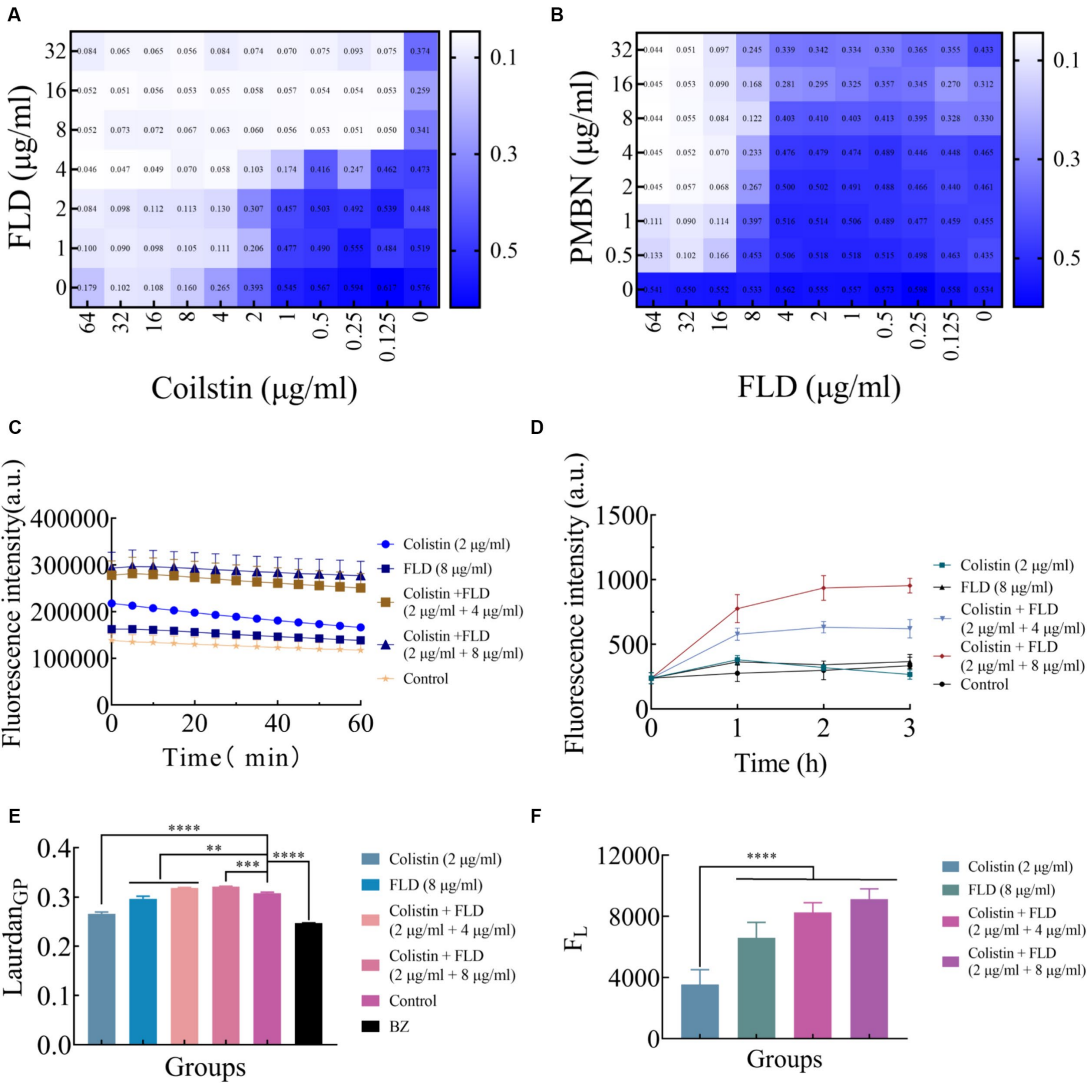
Colistin combined with FLD exert antibacterial effects through membrane. **(A)** Exogenous addition of LPS (10 μg/mL) had no effect on the antibacterial effects of colistin and FLD combination against KP2108. Measured by chequerboard microdilution assays. **(B)** The antibacterial effect of FLD was appeared in the presence of 10 μg/mL PMBN as an outer membrane permeabilizer. **(C)** Increased membrane permeability after treatment of colistin (2 μg/mL) combined with FLD (4 and 8 μg/mL). The membrane permeability was determined by NPN, a small molecule that cannot effectively cross the outer membrane. **(D)** The dynamic curves of the integrity of inner membrane probed with PI for KP2108, under the treatments of colistin (2 μg/mL), FLD (8 μg/mL), and colistin (2 μg/mL) combined with FLD (4 and 8 μg/mL). **(E)** The fluidity of membrane was decreased for KP2108 after treatment of colistin (2 μg/mL) and FLD (4 and 8 μg/mL). BZ was used as the positive control. **(F)** Colistin (2 μg/mL) and FLD (4 and 8 μg/mL) induced membrane depolarization, demonstrated by the fluorescence leakage from KP2108, when compared with colistin treatment. **p<0.01, ***p<0.001, ****p<0.0001.

The integrity and functionality of the inner membrane are essential for the bacterial survival and growth (Pader et al., 2016; Ye et al., 2021). After treated with colistin and FLD combination, disruption of the membrane structure allowed PI to enter cells, prompting an evaluation of outer and inner membrane integrity following different treatments. The primary distinction between Gram-positive and Gram-negative bacteria lies in their cell wall structural components. As LPS is a main component of the OM, the impact of LPS on the antibacterial activity of FLD was assessed. The exogenous addition of LPS had a negligible influence on the activity of FLD (Figure 3A). The membrane permeabilizers PMBN enhanced the antibacterial activity of FLD against KP2108 (Figure 3B), indicating FLD’s strong potential as an adjuvant for colistin. The permeability of the outer membrane was evaluated in the treated bacterial cells compared to the non-treated cells by detecting the fluorescence of NPN, which became strongly fluorescent upon binding to a phospholipid bilayer. The results indicated that colistin or FLD had a minimal effect on OM permeability, whereas the combination of colistin with FLD rapidly increased the OM permeability (Figure 3C). In addition, treatment with colistin combined with FLD disrupted the integrity of the inner membrane, as evidenced by the increased uptake of PI (Figure 3D). These observations aligned with the CLSM and flow cytometric analysis results, indicating permeabilization of both the outer and inner membrane. These results confirmed that colistin combined with FLD not only disrupted the integrity of the outer membrane but also damaged the inner membrane. Membrane fluidity and depolarization were also assessed. In the presence of colistin combined with FLD, membrane fluidity was significantly decreased (Figure 3E), and the cell membrane was depolarized, led to the leakage of fluorescence (Figure 3F).

### Mechanism of FLD against MDR *K. pneumoniae*

**Figure 4:**
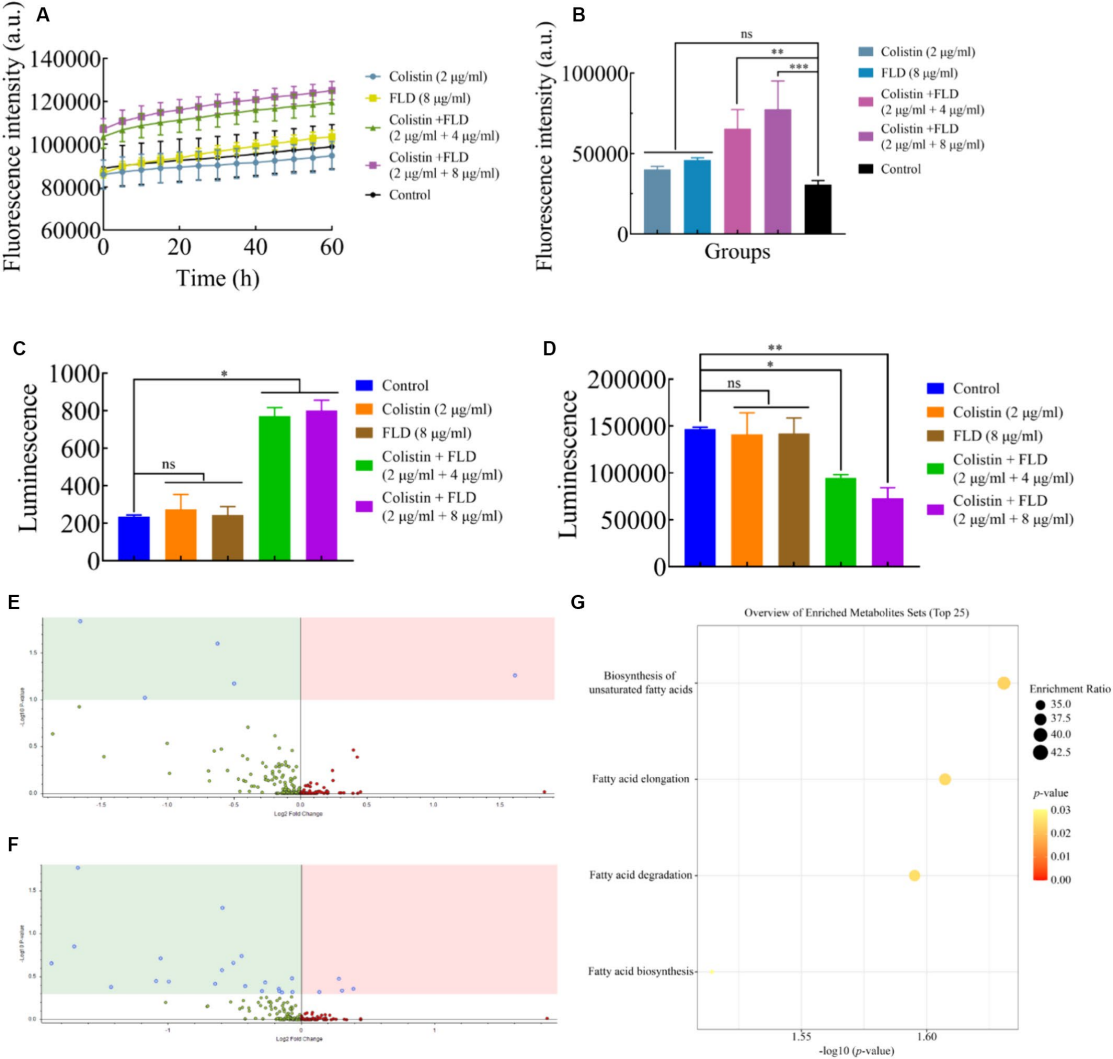
Mechanism of colistin in combination with FLD. **(A)** ΔpH was determined in KP2108 after treatment with colistin (2 μg/mL), FLD (8 μg/mL), and colistin (2 μg/mL) combined with FLD (4 and 8 μg/mL). **(B)** Accumulation of ROS in KP2108 treated with colistin (2 μg/mL), FLD (8 μg/mL), and colistin (2 μg/mL) combined with FLD (4 and 8 μg/mL). **(C,D)** Levels of extracellular ATP **(C)** and intracellular ATP **(D)** in KP2108 after treatment with colistin (2 μg/mL), FLD (8 μg/mL), and colistin (2 μg/mL) combined with FLD (4 and 8 μg/mL). **(E,F)** Volcano plot analysis of DEMs in KP2108 after exposure to colistin (16 μg/mL) or the combination of colistin (16 μg/mL) and FLD (16 μg/mL) for 1 h. **(E)** Negative ion model, **(F)** positive ion model. **(G)** KEGG enrichment analysis of DEMs after treatment with colistin or the combination of colistin and FLD. *p<0.05, **p<0.01, ***p<0.001, ****p<0.0001.

The change in membrane rigidity was demonstrated to disrupt bacterial homeostasis, leading to fundamental metabolic perturbations, including the dissipation of proton motive force (PMF) (Radlinski et al., 2019). Since membrane fluidity was reduced and proton leak was increased after treatment with colistin combined with FLD, ΔpH as one of the important parameters for PMF was measured with pH-sensitive probe BCECF-AM (Farha et al., 2013). Significant disruption in ΔpH was detected after treatment with both combinations (2 μg/mL colistin, 4 and 8 μg/mL FLD) compared to the control group, while no significant increase was observed after treatment with colistin (2 μg/mL) or FLD (8 μg/mL) (Figure 4A). Disruption of membrane homeostasis instigates the accumulation of ROS caused by bactericidal antibiotics (Kohanski et al., 2007). The results indicated that colistin or FLD did not affect significantly ROS accumulation. However, the ROS concentration was significantly enhanced after treatment with both compounds in combination, corresponding to membrane damage aggravated, further bacterial homoeostasis paralyzed (Song et al., 2020). The ROS level increased with the rise in compound concentration (Figure 4B). The disruption of PMF also can interfere with ATP levels (Van Acker and Coenye, 2017; Le et al., 2021). No significant differences were found in extracellular and intercellular ATP levels after treatment with colistin or FLD. However, colistin combined with FLD significantly increased extracellular ATP levels and decreased intercellular ATP levels in a dose-dependent manner (Figures 4C,D).

To further elucidate the molecular mechanisms by which FLD increased susceptibility observed in *K. pneumoniae* to colistin, the metabolome was analyzed using an untargeted metabolomics approach with UPLC-ESI-MS platform in both negative and positive modes. The strain KP2108 was treated with colistin or colistin-FLD for 1 h. Comparison of treatment with the combination and colistin revealed 29 differentially expressed metabolites (DEMs) (Figures 4E,F). Kyoto Encyclopedia of Genes and Genomes (KEGG) enrichment analysis demonstrated significant effects on biosynthesis of unsaturated fatty acids, fatty acid elongation, fatty acid degradation, and fatty acid biosynthesis in *K. pneumoniae* (Figure 4G). Taken together, FLD synergized with colistin by acting together on the membrane structure, inducing oxidization injury, and potentiate the antimicrobial effects of colistin.

### Homology modeling and docking analysis

**Figure 5:**
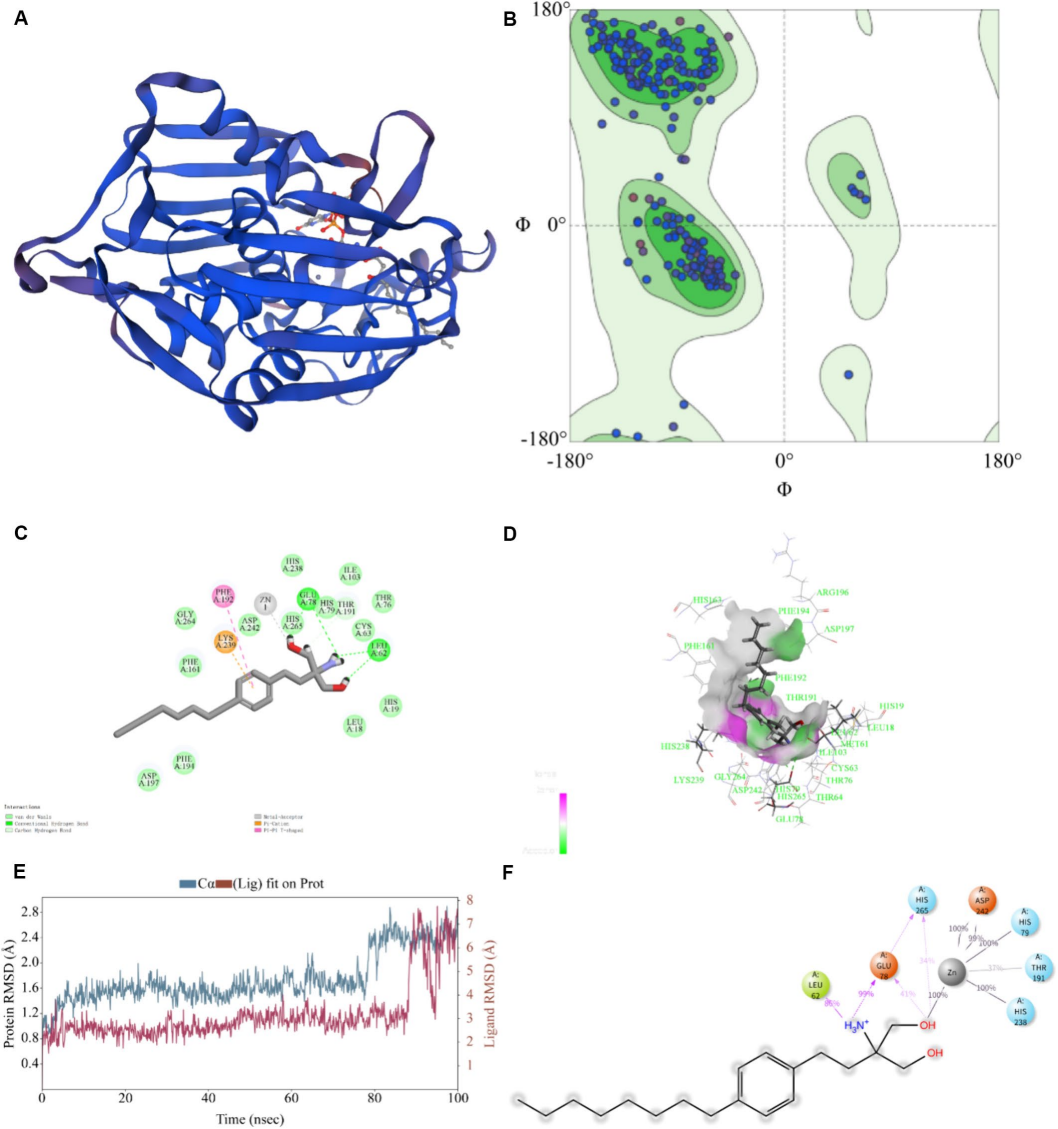
Prediction for binding model of FLD with LpxC protein (KPLpxC) and MD simulation. **(A)** The 3D model of LpxC protein for *K. pneumoniae* was established by using PDB: 4MDT template through homology modeling, 3D:3 dimension. **(B)** 3D quality of KPLpxC protein was assessed according to the Ramachandran plot (96.64%). **(C,D)** Interaction of FLD with KPLpxC protein, **(C)** two-dimensional, **(D)** three-dimensional. **(E)** Root mean squared deviation (RMSD) of Cα atoms and FLD in the KPLpxC-FLD versus simulation time. **(F)** The interaction force and force frequency of FLD to KPLpxC protein.

Typically, the sequence identity, QMEANDisCo, and GMQE scale range from 0 to 1 to indicate the credibility of the model reliability (Ma et al., 2020). The model result was seen in Figure 5A. For the KPLpxC protein compared with LpxC protein of *E. coli*, their sequence similarity was 95.74%, with a QMEANDisCo global score of 0.89 ± 0.05, and a GMQE value of 0.95. The Ramachandran Plots (96.64%) indicated good overall stereochemical quality of the generated model, conforming to protein stereochemistry rules (Figure 5B). Therefore, these results suggested high credibility of KPLpxC protein model.

As shown in Figures 5C,D, -CDOCKER ENERGY value was 48.585, which indicated the bound energy of the best docking position. The putative FLD binding site revealed interactions such as hydrogen bounds with GLU78 and LEU62, π-cation and π–π cation interactions with LYS239 and PHE192 and an interaction with the zinc ion. These non-covalent interactions promoted high-affinity bond of FLD to KPLpxC protein.

The MD simulations further validated the molecular docking findings. The root mean square fluctuations (RMSF) can respond to the flexibility of the protein during molecular dynamics simulation (Hadden et al., 2018). The RMSF of KPLpxC protein finally stabilized at about 2.4 Å, while the RMSF value of FLD ranged between 6.0 and 7.0 Å during the simulation (Figure 5E). The stable bond conformation was formed after MD simulations. During MD simulations, the interaction forces between FLD and KPLpxC protein were formed with hydrogen bonds for GLU78 at high frequencies of 99 and 41%, respectively. This illustration implied that GLU78 had a key role in FLD binding to protein (Figure 5F). In addition to these, other hydrogen bonds and electrostatic interactions also contributed to the combination. Therefore, FLD and the protein were optimized in the original bound conformation by kinetic simulation to make the conformation more stable, and have high affinity in this conformation.

### *In vivo* efficacy of colistin combined with FLD

**Figure 6:**
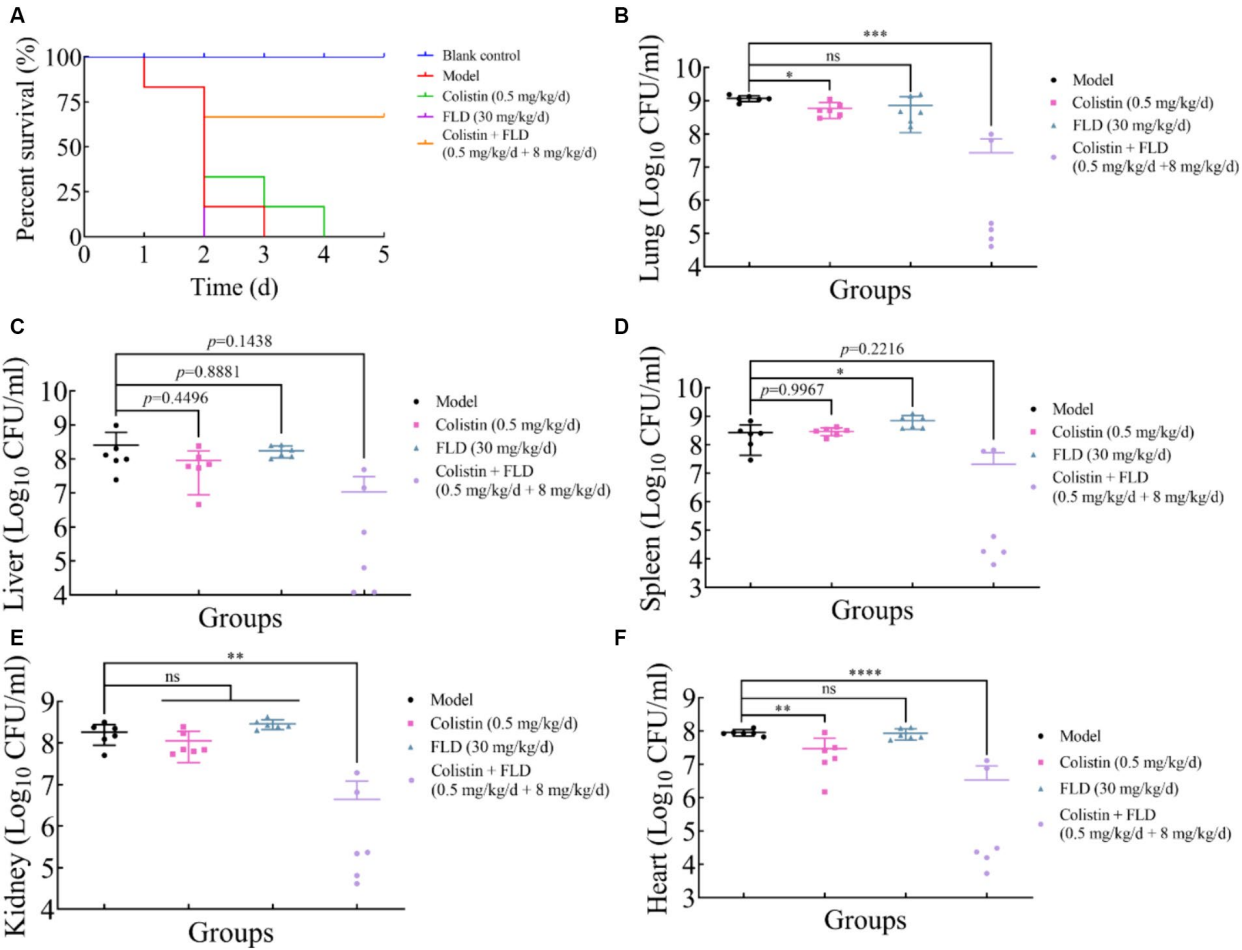
Fingolimod combined with colistin can treat infection from hypervirulence *K. pneumoniae* in mice. **(A)** Survival rates of female BALB/c mice (6 per group) infected with *K. pneumoniae* in treatment with colistin (0.5 mg/kg/d), FLD (30 mg/kg/d), and colistin (0.5 mg/kg/d) combined with FLD (8 mg/kg/d). **(B–F)** Bacterial loads in infected lung, liver, spleen, kidney, and heart in mice (6 per group). *p<0.05, **p<0.01, ***p<0.001, ****p<0.0001.

**Figure 7:**
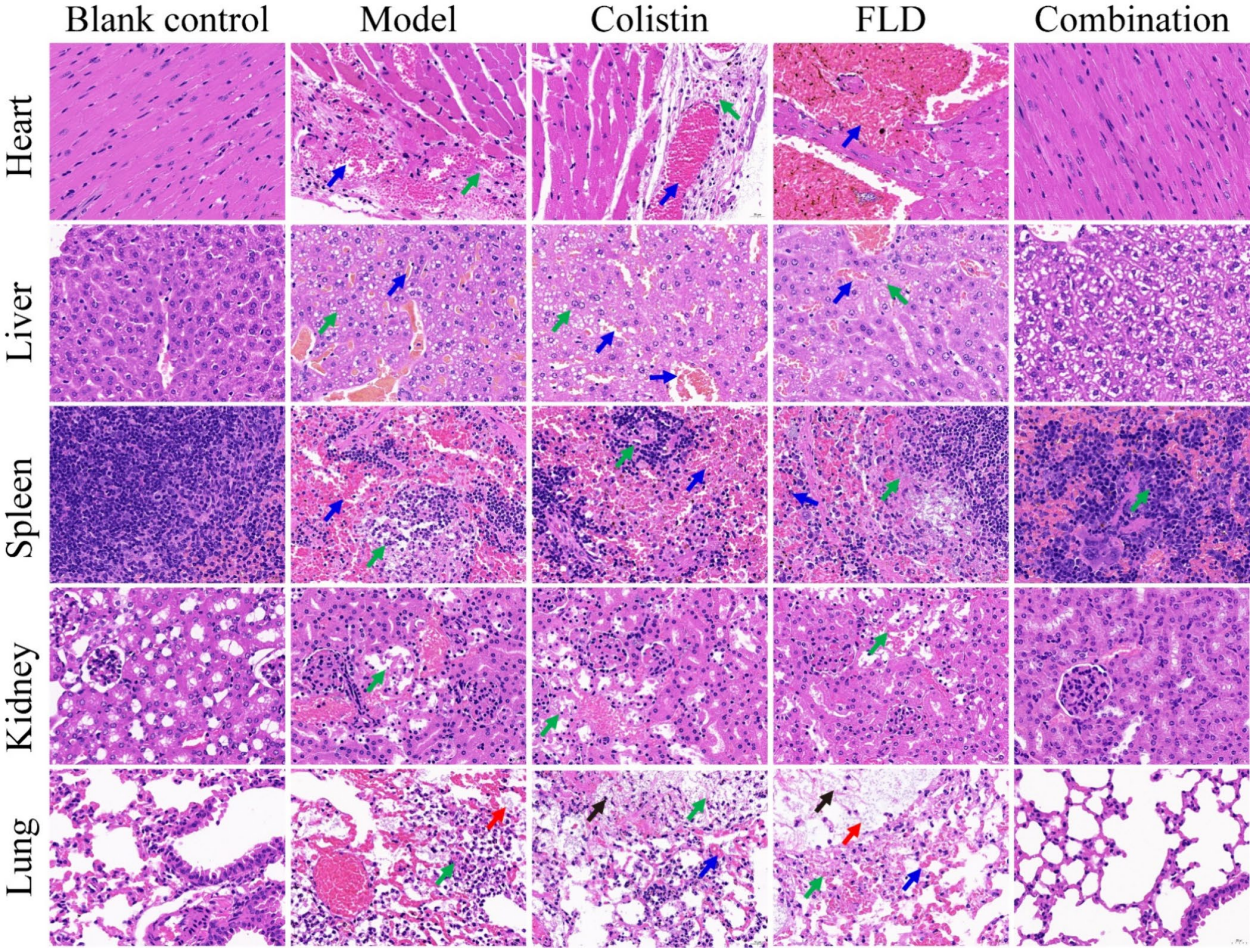
Histopathological analysis of different tissues using hematoxylin–eosin (HE) staining. The heart, liver, spleen, lung, and kidney were histological analysis in the mouse pneumonia model. Scale bar, 100 μm.

The *in vitro* results provided confidence to evaluate the anti-infective effectiveness of colistin synergized with FLD in a mouse pneumonia model. After establishing a lethal mouse model of pneumonia, the mice were grouped and treated. Three groups, with sterile saline, colistin, or FLD, resulted in all mice dying within 4 days. However, the survival rate in the combination group was higher than that in the single drug group (Figure 6A). Additionally, the bacterial load in different tissues of mice was reduced under combination treatment (Figures 6B–F). Moreover, the typical pathological changes in the infected organs were greatly attenuated as well (Figure 7). Compared with the model and single drug groups, the myocardial tissue in the combination group showed neat cardiac muscle fibers, however, edema, degeneration, necrosis, and cardiac hemorrhage were not observed. There were no histopathological alterations in the liver and the normal histological structure of the central vein and surrounding sinusoids were observed. The pathological changes on white pulps of spleen were recovered and lymphocytes were increased, widespread necrosis in red pulps were almost disappeared after combined treatment. Renal tubular dissolved and necrosis was significantly improved. After treatment with FLD and colistin, the alveolar epithelium revealed remarkable improvement in the lung, and the bacteria was not detected. These findings indicated the potential effectiveness of the combination treatment in treating pneumonia in mouse model.

## Discussion

ESKAPE strains (*Escherichia coli*, *Staphylococcus aureus*, *Klebsiella pneumonia*, *Acinetobacter baumannii*, *Pseudomonas aeruginosa*, and *Enterococcus faecium*) emerged as a significant global health concern due to their resistance to many antibiotics (Zhen et al., 2019). The acquisition of the plasmid-encoded colistin resistance gene *mcr-1* by these strains complicated treatment and imposed a substantial economic burden (Liu et al., 2016; Paterson and Harris, 2016; Portes et al., 2022). The side effects of colistin, especially nephrotoxicity and neurotoxicity, limited its clinical application (Vardakas et al., 2018; El-Sayed Ahmed et al., 2020). Consequently, there is a burgeoning interest in identifying synergistic adjuvants for colistin to solve the problem of MDR bacteria (Kim and Eom, 2021). Although FLD exhibits potent antibacterial activity on Gram-positive bacteria, its bactericidal activity on Gram-negative bacteria was not strong (Zore et al., 2021). FLD as an antibiotic adjuvant was rarely studied to enhance the efficacy of antibiotics. Therefore, this study aims to investigate the potential of colistin combined with FLD in treating *K. pneumoniae* infections with a validated *in vivo* pneumonia model.

The FLD had no bacteriostatic and bactericidal properties *in vitro*. However, FLD demonstrated a significant ability to enhance the *in vitro* and *in vivo* antibacterial activities of colistin, even at low concentrations of 8 μg/mL. These synergistic effects were verified in clinical *K. pneumoniae* strains derived from both human and animal source. The antibacterial activity of FLD in the presence of the outer membrane permeabilizer PMBN indicated that the outer membrane act as a physical barrier to restricts FLD entry into intracellular space. The integrity of physical and functional cytoplasmic membrane is essential to cell survival (Hurdle et al., 2011). The approval and marketing of FLD raise the possibility that it lacks a significant cytotoxic effect. Moreover, FLD had been considered to have no potential to induce resistance in *S. aureus* after continuously cultured on FLD-supplemented medium for 20 days (Gilbert-Girard et al., 2020). Therefore, it is recommended that FLD can be used in combination with standard-of-care antibiotics to enhance their antibacterial activity.

The outer membrane permeability was enhanced after treatment with colistin, attributed to the formed complex between colistin and LPS, disrupting the stability of the outer membrane (Saikia et al., 2022). However, colistin treatment had no significant impact on inner membrane integrity, which was only compromised on combined treatment with colistin and FLD. This observation suggested that FLD was able to traverse the outer membrane in the presence of colistin. Following treatment with both colistin and FLD, a decrease in membrane fluidity with significant cellular surface deformation was observed. This phenomenon may be linked to a decrease in the proportion of unsaturated fatty acids in the cell membrane, coupled with an increase in saturated fatty acids (Álvarez-Ordóñez et al., 2009). This membrane fluidity results corresponded to the change of metabolome, both of which had an impact on bacterial fatty acid metabolism. An intricate relationship was found between the fatty acid and LPS biosynthetic (Emiola et al., 2016). The majority of proteins involved in the fatty acid elongation cycle were found in the LpxC interactome, indicating a close relationship between LPS and phospholipid (PL) biosynthesis (Thomanek et al., 2019). *K. pneumoniae* has tight coordination with inner and outer membrane biosynthesis via FabZ and LpxC (Mostafavi et al., 2018). A 3D structure of KPLpxC protein was constructed and a 100 ns MD simulation was carried out to validate the reliability of the docking result between FLD and KPLpxC. The docking analysis revealed a strong binding affinity between FLD and KPLpxC and the complex displayed robust conformational stability following optimization of the initial conformation. The binding site of FLD differed from that of LPC-058 (an inhibitor of LpxC protein) (Lemaitre et al., 2017). This discrepancy may be attributed to the protein genus as the *lpxC* gene was conserved in Gram-negative bacteria (Lee et al., 2011). Despite this difference, the binding pockets of FLD and LPC-058 exhibited similarity, leading to strong binding interactions with LpxC protein. Intermolecular forces were formed between FLD and KPLpxC to contribute their bond and form a compact complex.

The disruption of the cytoplasm membrane resulted in a series of metabolic dysregulation (Radlinski et al., 2019). ROS in the bacteria was probably involved in the association between apoptotic biomarker and stress-mediated bacterial death. However, the precise relationship between ROS alterations and bacterial death remained ambiguous (Hong et al., 2019). The escalation of ROS accumulation may serve as the ultimate trigger for bacterial demise. ROS was thought to cause a variety of intracellular damage, such as lipid peroxides, carbonylated proteins, and DNA damage (Dwyer et al., 2015). Compared with the untreated, colistin, and FLD treatment groups, a significantly increase in ROS accumulation was observed following the treatment with colistin and FLD. Excessive ROS production and ROS-mediated cell damage led to severe membrane damage, including depolarization and reduced fluidity. This phenomenon may be attributed to ROS-induced lipid damage after the colistin-FLD treatment, altering membrane conformation and tension. Meanwhile, intracellular ATP levels exhibited a considerable decrease in the colistin-FLD treatment. There resulted from several factors. For example, the disruption of bacterial cell membrane and the insufficient generation of ATP for its essential consumption could cause the ATP loss. The increased concentration of extracellular ATP levels indicated membrane disruption and intracellular ATP leakage. The preliminary findings suggested that colistin combined with FLD destroyed bacterial membranes, caused leakage in the bacterial cell membrane.

The LD_50_ in mice was 25 mg/kg when colistin was injected intraperitoneally. Although colistin has been reported to be nephrotoxic, nephrotoxicity did not occur after 1 week of injection at concentrations below the LD_50_ (Nord and Hoeprich, 1964). FLD was approved for marketing at 0.5 mg/day by FDA. In a 14-day study of 5 mg/day in the human body, FLD was not associated with impaired oxygenation or oxygen desaturation with exercise or an increase in airway responsiveness to methacholine. Subjects on FLD treatment had a normal bronchodilator response to inhaled beta-agonists (FDA, 2010). 8 mg/kg/day of FLD in the mice was calculated on body surface area as equivalent conversion to 0.88 mg/kg/day for a 70-kg human being. In this study, the mice in the combination group were treated with FLD at 8 mg/kg and colistin at 1 mg/kg, therefore, it can be deduced that the efficacious combination is safe in mice at the used doses. Certainly, further investigation is needed to be carried out. As the combination of colistin with FLD in infected group showed better survival rates than the infected group treated with colistin, combination in colistin and FLD can be used to cure severe infection caused by hypervirulent *K. pneumoniae*. Although significant synergistic effect was observed between colistin and FLD, caution is advised regarding their combined clinical use for FLD as an immunosuppressor. Further investigation into the molecular structure of FLD as the modification of major chemical functional groups is warranted to mitigate its potential side effects of clinical applications.

## Conclusion

The FLD exhibited efficacy in reversing the resistance of *K. pneumoniae* to colistin at the concentration of 8 μg/mL. The co-administration of FLD and colistin caused significant disruption of the inner membrane in MDR *K. pneumoniae* and influenced fatty acid metabolism. FLD demonstrated inhibitory potential toward the *K. pneumoniae* LpxC protein through its strong binding affinity and maintenance of structure integrity. *In vivo* studies in mouse models confirmed the synergistic efficacy of colistin and FLD in reaching to 67% of the survival rate and relieving pathological damage. Overall, FLD showed a promise as a valuable adjuvant to colistin in the treatment of MDR *K. pneumoniae* infections. Nevertheless, further research is necessary to elucidate the precise mechanism of action, specific binding targets, and pharmacodynamics of FLD.

## Data availability statement

The raw data supporting the conclusion of this article will be made available by the authors, without undue reservation.

## Ethics statement

The animal study was approved by Lanzhou Institute of Husbandry and Pharmaceutical Sciences of CAAS. The study was conducted in accordance with the local legislation and institutional requirements.

## Author contributions

XG: Data curation, Formal analysis, Methodology, Software, Writing – original draft, Writing – review & editing. Z-DZ: Formal analysis, Methodology, Writing – review & editing. Y-XL: Methodology, Writing – review & editing. R-CH: Methodology, Writing – review & editing. Y-JY: Data curation, Formal analysis, Writing – review & editing. X-WL: Funding acquisition, Project administration, Writing – review & editing. J-YL: Funding acquisition, Investigation, Writing – review & editing.

## Footnotes

^1^ http://www.rcsb.org/pdb

^2^ http://swissmodel.expasy.org/
